# On Going to a New Era of Microgel Exhibiting Volume Phase Transition

**DOI:** 10.3390/gels6030026

**Published:** 2020-08-17

**Authors:** Haruma Kawaguchi

**Affiliations:** Faculty of Science and Technology, Keio University, Hiyoshi, Yokohama 241-0814, Japan; haruma@mvg.biglobe.ne.jp

**Keywords:** microgel, volume phase transition (VPT), VPT temperature, precipitation polymerization, co-non-solvency, poly(N-isopropylacrylamide), hydrophobic hydration, hard-core soft-shell microsphere, electrophoretic mobility

## Abstract

The discovery of phenomena of volume phase transition has had a great impact not only on bulk gels but also on the world of microgels. In particular, research on poly(*N*-isopropylacrylamide) (PNIPAM) microgels, whose transition temperature is close to body temperature, has made remarkable progress in almost 35 years. This review presents some breakthrough findings in microgels that exhibit volume phase transitions and outlines recent works on the synthesis, structural analysis, and research direction of microgels.

## 1. Introduction

This article reviews the development of research on microgels exhibiting volume phase transition (VPT) which was discovered by T. Tanaka [1]. The preparation of the microgels, physicochemical characteristics of the microgels/dispersions, and future prospect of the microgels are discussed. There are various microgels exhibiting VPT, but this review focuses on poly(acrylamide derivatives), especially PNIPAM. 

The contents of this review are as follows:

Section 1 is an introduction and presents the definition of some terms related with thermosensitive microgels.

Section 2 describes “precipitation polymerization”, the most popular method for making thermosensitive microgels, and introduces the characteristic points of the products.

Section 3 presents the things to consider when designing thermosensitive microgels, which include phase transition temperature, particle size, degree of cross-linking, and compounding.

Section 4 presents physicochemical characteristics of thermosensitive microgels; especially, surface tension, electrophoresis, and dispersion stability are discussed. 

Section 5 presents guidelines for preparing effective thermosensitive microgels, and Section 6 presents conclusions. 

References [1,2,3] are representative reviews on this subject [2,3,4].

### 1.1. Definition of Nanogel and Microgel

Gels are a soft material having a network structure that holds a large amount of liquid or gas. Generally, the gels can keep their own form even after massive adsorption due to cross-linking points distributed throughout. The components of gels are not limited to organic ones, but there are some inorganic gels such as silica gel, clay, and hybrid gels. This review involves organic gels, which are mainly gels that can hold a large amount of water: so-called hydrogels. They are used in a variety of fields such as food, hygiene materials, medical supplies, and cosmetic industries.

**Figure 1:**
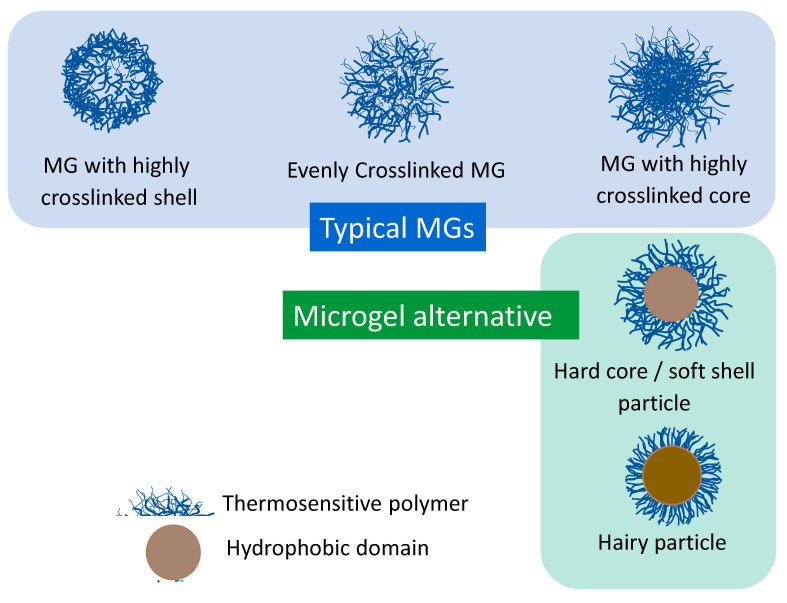
Microgels (MG) and their alternatives.

**Figure 2:**
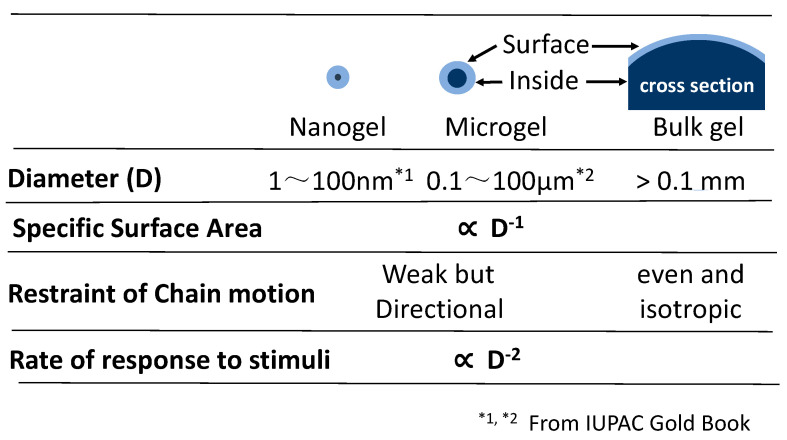
Comparison among nanogel, microgel and bulk gel.

Fine particulate gels (Figure 1, Typical MG) were referred to as nanogel or microgel, depending on their size (Figure 2). Compared to microgels, nanogels have a volume/(surface area) ratio that is 1 to 2 orders of magnitude smaller, the effect of volume on physical properties is small, and they are not suitable for discussing volume phase transitions Therefore, in this review, discussions are mainly focused on microgels.

Sometimes, hard-core and gel-shell microspheres (Figure 1, Microgel alternative) are used as alternatives of microgels for some purposes because the structure of their surface layer (shell) is similar to that of microgels. Star polymer is considered to be a unique nanogel having only one cross-linking point in the center. In addition, hairy microspheres (Figure 1, bottom right) may be regarded as a kind of core–shell microspheres, in which the core itself corresponds to a single big cross-linking point and hairs with unrestrained ends show rapid and sharp response to external stimuli.

These microspheres are regarded as “microgel” and discussed in this review.

### 1.2. Comparison of Microgels and Bulk Gels 

As shown in Figure 2, bulk gels are composed of a large volume of interior polymer with a very small amount of polymer exposed to the surface. Therefore, the property of bulk gels is mostly controlled by the inner component, which suffers un-neglectable isotropic pressure. 

The physical condition of microgels is quite contrary to that of bulk gels. In microgels, a significant proportion of polymer chains is exposed to the medium (water) in the case of hydrogel, and it diverges their stress to the medium. Therefore, the state of polymer chains changes quickly within a short distance from the core to the surface. The huge difference of the chain states in a microgel is reflected in their complicated response.

One of the significant differences between bulk gels and microgels is the response speed to stimuli. The speed changes inverse proportionally to the square of gel size. A 100 nm diameter micro-hydrogel adsorbs water 100 times faster than a 1 micron diameter microgel. This is the reason why a disposable diaper is composed of loosely aggregated microgels. 

### 1.3. Volume Phase Transition of Microgels

Without referring to molecular dynamics, here, we will interpret the volume phase transition conceptually.

Polyacrylamide is a hydrophilic polymer. The molecule is composed of a hydrophilic portion (amide group) and a hydrophobic portion (main chain –C–C-), in which the former is more prevalent than the latter. In contrast, some N-substituted acrylamide polymers, such as poly(N-isopropyl-acrylamide) (PNIPAM) and poly(N-n-propyl-acrylamide), etc. have subtly balanced hydrophilic and hydrophobic portions. The microgels composed of these polymers in water are hydrated and swollen below a certain temperature. Below that temperature, water molecules surround hydrophobic polymer chains in order to decease the total interfacial energy of a gel–polymer/water system. Namely, an entropy loss of water molecules surrounding the hydrophobic polymer chain is cancelled by enthalpy gain due to hydrogen bonding between water molecules surrounding hydrophobic parts of polymers. This interaction is called hydrophobic hydration, which is necessary to keep hydrophobic materials in aqueous medium. 

When the microgel dispersion is warmed up above a certain temperature, hydrophobic hydration fluctuates and water molecules leave polymer chains. The dehydrated polymer chains form globules. The dehydration and globule formation occur collaboratively, and as the result, definite volume phase transition is observed in a very narrow temperature range. This characteristic temperature is called the volume phase transition temperature (VPTT) or lower critical solution temperature (LCST) [5,6]. 

The transition of polymers at the LCST can be confirmed spectroscopically as follows; FTIR absorption assigned to C=O…H-O-H (hydration of the polymer chain) appears below the LCST and C=O…H-N (polymer chain interaction/globule formation) appears above the LCST [7,8].

These spectral data prove deswelling and shrinkage of the microgel. The thermoresponsive behavior and microenvironment of PNIPAM microgels were also studied by the fluorescent label method [9].

The volume phase transition caused by hydration from/to dehydration of microgels is directly observed as a change in the hydrodynamic diameter or radii of microgel. Sbeih et al. studied the temperature dependence of the hydrodynamic radii of PNIPAM microgel by dynamic light scattering (DLS) and rheology experiments. The authors yielded the polymer volume fraction, φ_0_, by (Differential Scanning Calorimetry) DSC, which is associated with microgel particle elasticity. It increased with cross-link density, but the polymer volume fraction in the microgel at collapse, φ_collapsed_, which was obtained by rheology experiments, was independent of cross-link density [10]. 

Following instrument analysis, poly(N-substituted acrylamide)s’ LCSTs were studied using molecular dynamics simulations to solve a complex interplay between hydration and intermolecular interactions [11].

There are some polymers that show a phase diagram opposite to that of LCST-type polymers; that is, upper critical solution temperature (UCST)-type polymers, such as poly(allyl amine)-co-(allylureido), poly(3-dimethyl- (methacryloyloxyethyl ammonium propane sulfonate), etc. [12].

In this review, UCST is not discussed, so VPTT and LCST are used without distinction.

## 2. Synthesis of Microgels Exhibiting VPT

There are several reaction systems in which two reactions, polymerization and particle formation, proceed simultaneously. They include emulsion polymerization, soap-free emulsion polymerization, mini-emulsion polymerization, suspension polymerization, precipitation polymerization, dispersion polymerization, etc.

When selecting a polymerization method for producing desired particles, the following items are examined: chemical and physical properties of particles, technical rationality, environmental burden, cost, etc. Considering the impact on the environment, water is chosen as the reaction medium, and the additives are eliminated as much as possible. In view of this situation, precipitation polymerization in water is first selected for the preparation of microgels exhibiting VPT.

This review also includes the hard core–soft (gel) shell particles, considering that the characteristics of the microgel appear even if the core of the microgel is made of a temperature-insensitive hydrophobic polymer. The hard core–soft shell particles are generally prepared by soap-free emulsion polymerization, or if necessary, followed by graft polymerization.

### 2.1. Precipitation Polymerization Elucidated from Phase Diagram

A precipitation polymerization system is composed of only three materials: monomer (e.g., NIPAM), water (the temperature > LCST of PNIPAM), and an initiator. Here, hot water (e.g., 70 °C) is used as a solvent for NIPAM monomer but a non-solvent for PNIPAM.

It does not matter whether or not a cross-linking agent is added (the reason for it will be presented later). Other additives such as surfactants and stabilizers are not required. A simple polymerization recipe simplifies product purification. The polymerization starts with an aqueous monomer solution and ends with the formation of a microgel dispersion.

It is incorrect to call this system a soap-free emulsion polymerization system because the role of water is different between precipitation polymerization and emulsion polymerization.

The monomer in the emulsion polymerization is consumed in “monomer polymer particles”, and the consumed amount of monomer is supplied from “monomer droplets”. At this time, the aqueous phase delivers the monomer in the droplets to the monomer polymer particles, which is the place of polymerization. The water phase is not a place for polymerization. On the other hand, in the precipitation polymerization, the monomer is dissolved in the whole system, and the aqueous phase serves as a polymerization place. It is also unreasonable to call precipitation polymerization, as soap-free emulsion polymerization at extremely low monomer concentrations.

**Figure 3:**
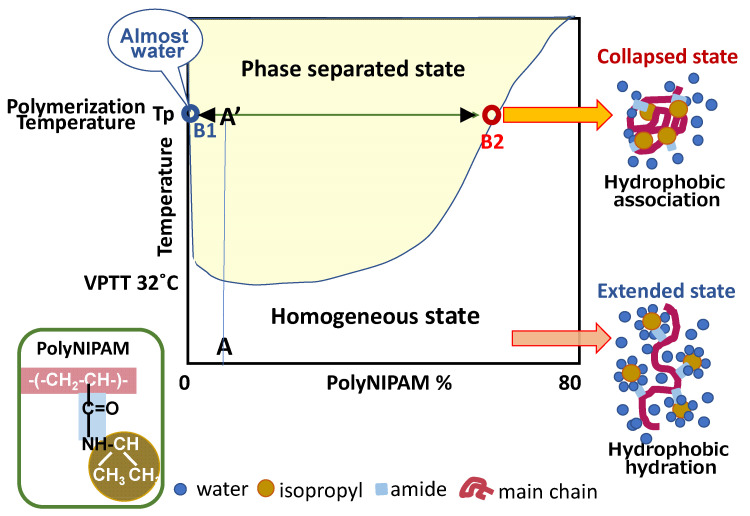
Phase diagram of PNIPAM/water system and state of PNIPAM molecule.

Figure 3 is a phase diagram of a poly-N-isopropyl acrylamide (PNIPAM)/water system (rewritten from the original one by M. Heskins, et al. [13]).

According to this phase diagram, the products of polymerization at Tp (polymerization temperature) do not exist at Point A’, but they separate to Point B1 (almost 100% water: dispersion medium) and Point B2 (dispersing collapsed microgels). The volume ratio of B2 to B1 is close to A’-B1/A’-B2. The collapsed microgels are composed of accumulated polymer chains having initiator-originated ionic groups at their chain ends. The microgels hold an appreciable amount of water [14]. Tauer et al. revealed that two kinds of water molecules are surrounding PNIPAM molecules in aqueous medium. One is water molecules strongly bound to PNIPAM and the other is water molecules that are loosely swelling PNIPAM. Their volume fractions at 40 °C are about 25% and 40–50%, respectively [15].

Figure 3 also informs us what happens when the microgels prepared by precipitation polymerization without a cross-linking reagent are cooled down to room temperature. Below VPTT, the microgels are expected to recover their hydrated structure, and they dissolve in water if the chain entanglement is not too tight. The system settles into a homogeneous solution where the polymer concentration is “A” in Figure 3. However, practically, a side reaction to form cross-links occurs during precipitation polymerization, and the microgel’s shape is retained. 

Before referring to specific polymerization examples, we outline the mechanism and kinetics of polymerization.

### 2.2. Mechanism and Kinetics of Precipitation Polymerization

The mechanism and kinetics of precipitation polymerization utilized in the preparation of microgels have not been thoroughly investigated. The McMaster group firstly studied the kinetics of polymerization of NIPAM in the presence of surfactant and suggested that the polymerization follows Smith–Evert Case 3 kinetics; that is, polymerization occurs in the particles having radicals greater than one [16]. In order to verify this concept, it seems necessary to confirm the distinction between particles and aqueous medium in the polymerization system.

Relatively recently, Richtering’s group worked to clarify the mechanism and kinetics of precipitation polymerization of NIPAM. They carried out the precipitation polymerization in a test tube without stirring in order to get reproducible results quickly. They found that the polymerization proceeds according to the following rate equation [17]:

Rp ∝ [M]^1.0^ [I]^0.5^

in which Rp is the rate of polymerization, [M] is the monomer concentration, and [I] is the initiator concentration.

This means that the place of precipitation polymerization is the aqueous medium, and the polymerization proceeds in the mode of solution polymerization 

The polymerization temperature is usually set ca. 30 degrees higher than the LCST of the target polymer. The choice of this condition is based on the strategy to get efficient phase separation between the polymer phase and water phase. A water-soluble initiator with a 10-h half-life of several hours is used to initiate the polymerization. Persulfate just meets the condition for the polymerization at 70 °C.

**Figure 4:**
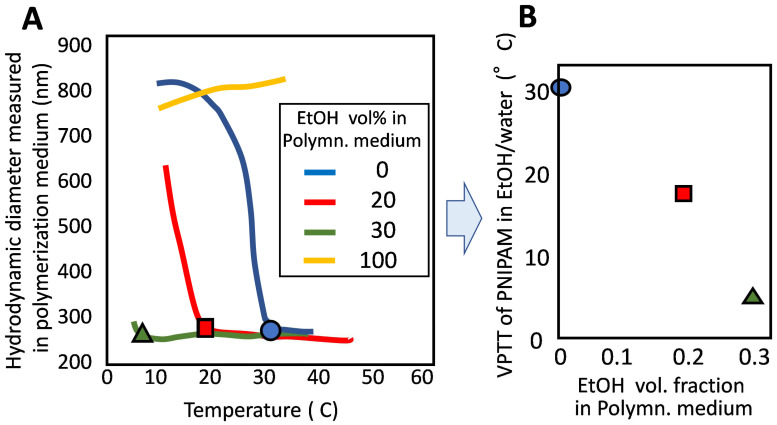
Temperature dependence of hydrodynamic size of microgel in polymerization medium (**A**), and VPTT of PNIPAM in mixed solvents with different compositions (**B**).

However, sometimes, polymerization at a lower temperature is requested to suppress side reactions. In order to meet this demand, the first thing to do is to change the polymerization initiation system. Yet it might be not enough, because the polymerization at a temperature close to the VPTT of PNIPAM would loosen the phase separation between the polymer-rich phase and the aqueous phase. That is, lowering the polymerization temperature causes the reduction of the difference in composition B1 and B2. To avoid this, we considered using a co-non-solvent system, because polymerization in a co-non-solvent system proceeds keeping PNIPAM’s VPTT lower than the VPTT in water. There are some co-non-solvent systems for PNIPAM [18,19], and we chose a water–ethanol system. Thereby, it becomes possible to carry out the polymerization at a lower temperature while keeping an appreciable difference between the polymerization temperature and VPTT [20]. We confirmed that PNIPAM had a much lower VPTT in water/ethanol than 32 °C (VPTT in water) (Figure 4A,B). Then, we carried out the precipitation polymerization of NIPAM at 50 °C in the co-non-solvent, ethanol/water = 1/4. Monodisperse and stable microgels were obtained not only in a flask but also in a silica tube with 2 μm diameter. A one-dimensional array of PNIPAM microgel was obtained from the latter non-stirring system in the tube [20].

### 2.3. Synthesis of Microgels and Their VPT Behavior

#### 2.3.1. Microgels Prepared by Precipitation Polymerization and Their VPT

Here is a typical recipe and result of precipitation polymerization of NIPAM reported by Pelton et al. [21]:

Typical recipe: NIPAM 14 g/L,

methylene-bis-acrylamide (MBA, cross-linker) 1.4 g/L,

potassium persulfate (initiator) 0.83 g/L,

water 720 mL,

polymerization temperature: 70 °C.

The thermosensitivity of the obtained PNIPAM microgels was confirmed by an absorbance of microgel dispersion versus temperature curve. The curve showed an inflection point around 34 °C that corresponded to the critical coagulation temperature of the microgel. 

According to TEM, the diameter of dried microgels was ca. 500 nm. The microgels formed a two-dimensional array, keeping a constant interparticle space on the TEM. The formation of an ordered array structure is a proof of the formation of monodisperse microgels [2]. 

The electrophoretic mobility of the above-mentioned PNIPAM microgels was measured in order to clarify the relation among the temperature, volume of the microgels, and charge density of the microgels. With increasing temperature, the microgels shrunk, while the charge density and electrophoretic mobility increased [22]. All the changes were discontinuous due to the volume phase transition.

Various N-substituted acrylamides other than NIPAM were used for precipitation polymerization, and the copolymerization of them with NIPAM was also performed to confirm the thermosensitivity of the resulting microgels. When the polymers have a similar reactivity to each other, random copolymers are usually obtained, and the LCSTs of the copolymers are the average between the two. If the comonomers have different monomer reactivity constants, the copolymers show a stepwise volume phase transition, even if the comonomers are batch-wisely put into the reactor.

The sequential precipitation polymerization of two N-substituted acrylamide monomers gives core–shell microgels in which a core microgel is formed first and shell layer formation follows [23].

Richtering et al. prepared PNIPAM–core and PNIPMAM (poly(N-isopropylmethacrylamide))–shell microgels and analyzed the structure with SANS. The core–shell microgel displayed two-step shrinking behavior [24]. According to detailed study on the core–shell interface, the core and shell demonstrate mutual influence; for example, the thick PNIPMAM shell did not allow the transition of the PNIPAM core [25,26].

In contrast, copolymer microgels consisting of poly(*N*,*N*-diethylacrylamide) (PDEAAM, LCST: 37 °C) and PNIPAM (LCST:32 °C) presented an unusual dependence of LCST on comonomer composition [27]. That is, a copolymer microgel with nearly equimolar composition presented LCST that was lower than those of both corresponding homopolymer microgels. This phenomenon was attributed to the strong hydrogen bonding between the DEAAM and NIPAM.

Another exceptional behavior was observed in PNIPMAM (VPTT: 45 °C)–core and poly(*N*-n-propylacrylamide) (PNnPAM) (VPTT; 23 °C)–shell microgels [28]. Although they were prepared in sequential polymerization, core and shell components displayed gradient profiles with strong interpenetrations. Interestingly, the cores embedded in shells were bigger than their isolated core-only precursor particles at temperatures intermediate to both transition temperatures. The phenomenon suggested promoted swelling of the core polymer, which leads to a linear swelling of the material.

Lyon’s group and Richtering’s group studied the effect of microlgel coupling on VPTT, independently. The former used PNIPAM and poly(NIPAM-co-AAc) microgels, etc. [29] and the latter used some core–shell microgels such as PNIPAM core–PDEAAM shell microgel [30]. In the latter, the amount of intramolecular hydrogen bonds in the following 5 kinds of microgels were compared: (1) PNIPAM microgel (MG), (2) NIPAM_0.55_–DEAAM_0.44_ copolymer MG, (3) PNIPAM_0.25_–core–PDEAAM_0.75_–shell MG, (4) PDEAAM_0.80_–core–PNIPAM_0.20_–shell MG, and (5) PDEAAM MG. Note that PNIPAM carries a donor (-NH) and acceptor (-C=O), whereas PDEAAM carries no donor. FTIR measurements showed that MG(2) possessed an extremely large amount of intramolecular hydrogen bonds and therefore showed volume phase transition at the lowest temperature.

#### 2.3.2. Other Techniques to Prepare Thermosensitive Microgels

There are several papers that report the synthesis of microgel by polymerizations other than precipitation polymerization.

PNIPAM microgels having an inhomogeneous internal structure were obtained by inverse microemulsion polymerization followed by seeded polymerization with different NIPAM/crosslinker ratios [31]. The microgels performed larger water uptake and faster swelling than conventional ones. This result would be explained by the elegant emulsification condition in a microemulsion polymerization system.

Inverse suspension polymerization—that is, polymerization of water-soluble monomer in a w/o system—was reported to give 10 μm microgels. The swelling–deswelling characteristics of the obtained microgels were similar to those prepared by conventional ways, albeit with differing particle sizes [32].

The use of microfluidic devices is a recent trend of microgel synthesis.

Precisely designed microfluidic devices demonstrated the ability for producing PNIPAM microgels with a uniform size and unique inner morphologies, such as microgels with embedded materials, microgels with multiple voids, etc. [33,34].

This technology is expected to secure an important position in microgel manufacture.

A PNIPAM–silica composite microgel was synthesized by a one-pot process of precipitation polymerization of NIPAM in supercritical carbon oxide. The resulting composite particles were about 100 nm in diameter [35].

Microgels can also be made from existing polymers. On raising the temperature of the dilute PNPAM solution, PNIPAM microgels were obtained by the phase-separated molecular integration method [36].

### 2.4. Synthesis and Characteristics of Microgel Alternatives

#### 2.4.1. Hard Core/Soft Shell Microspheres Exhibiting VPT

Core–shell particles whose core is hard polymer and shell is hydrogel are regarded as an alternative of microgels (Figure 1, bottom right), because the structure and property of the shell layer of core–shell particles are similar to those of the outer layer of microgels.

Soap-free emulsion copolymerization is a recommendable method to prepare core–shell particles from two (or more) monomers with different hydrophilicity, such as a couple of acrylamide and styrene (St). Here, St is insoluble in water, and polymerization starts from St/(acrylamide aqueous solution) emulsion.

In this polymerization, a hydrophilic monomer starts to polymerize first to form a nuclei of particle composed of hydrophilic monomer-rich polymers, but the hydrophilic polymers are not pushed in the core, because hydrophobic polymers made later burrow into hydrophilic polymers to minimize the interfacial energy of the system. The resulting particles have less hydrophilic polymer core and more hydrophilic polymer shell. For example, the soap-free emulsion polymerization of St and NIPAM gives a PSt-core PNIPAM-shell particles (even at 70 °C, PNIPAM is much more hydrophilic than PSt [13]) in which PNIPAM and PSt have the least interaction at the core–shell interface. When the second-shot of NIPAM is added to the core–shell particle dispersion, further developed core–shell particles are obtained [37]. 

Thus-obtained PNIPAM shell-carrying microspheres could be used as an alternative of PNIPAM microgels. Core–shell particles are advantageous over microgels in that they can be mass-produced at one time and, due to the hard core, deformation of the particles can be prevented.

Polymers of acryloyl pyrolidine (APr) and acryloyl piperidine (APp) have LCSTs of 55 °C and 5 °C, respectively. When APr and APp were copolymerized in aqueous medium at 4 °C at various APr/(Apr + APp), the series of copolymers showed cloud points (CP) that varied linearly with APr/(Apr + APp). Here, CP refers to the temperature at which the polymer solutions become cloudy.

In addition, PSt core (copolymer of APr and APp)–shell particles were prepared with different APp/(Apr + APp) by soap-free emulsion polymerization. Then, the flocculation temperature (temperature at which the dispersed particles lose their hydration protection and flocculate, FT) of each core–shell particle was measured to make a figure of FT versus Apr/(Apr + APp).

The CP and FT values were plotted versus Apr/(APr + APp). The plots of two series overlapped neatly. This result shows us that PSt core (copolymer of Apr and APp)–shell particles can be regarded as an alternative of thermosensitive microgels [38].

#### 2.4.2. Hairy Microspheres Exhibiting VPT

Hairy particles (Figure 1, right bottom) are another type of core–shell particles. Hairs are linear polymer chains overgrown from the core surface.

P(AAm-co-MBA) core–PNIPAM hair microgel was synthesized by grafting PNIPAM to the core (5–10 nm in diameter); contraction of a PNIPAM hair layer above 34 °C caused the squeezing out of water from core particles [39]. 

The PNIPAM brush on a core particle formed a hydrophobic nanocavity for the organic substrates, and it provided a suitable reaction microenvironment below and above the LCST of PNIPAM [40].

Unique hairy particles with a uniform length were formed by living polymerization, which starts from active sites on the existing particle surface. Here is an example of unique thermo-, pH- and ionic strength-sensitive hairy particles. The PSt core particles with diblock copolymer hairs composed of PNIPAM (N block) and PNIPAM-ran-poly(acrylic acid) (NA block) were prepared by sequential living radical graft polymerization using a photo-initiator. The hairy particles, (PSt core)–N–NA or (PSt core)–NA–N particles, showed a variety of response to temperature, pH, and ionic strength [41,42].

## 3. Design of Thermosensitive Microgels

When constructing thermosensitive microgels for applications, there are several essential items to be considered. Among them, the VPTT of the microgel, size of the microgel, and crosslink density are especially important.

### 3.1. VPTT and Its Sharpness of Microgels

The guidelines are presented for finding microgels with the expected VPTT by referring to the relationship between the molecular structure and VPTT.

As mentioned in Section 1.1, polymers exhibiting volume phase transition have a molecular structure including well-balanced hydrophiles and hydrophobes. Acrylamides and methacrylamides having various substituents provide the line-ups to meet the requirements.

**Figure 5:**
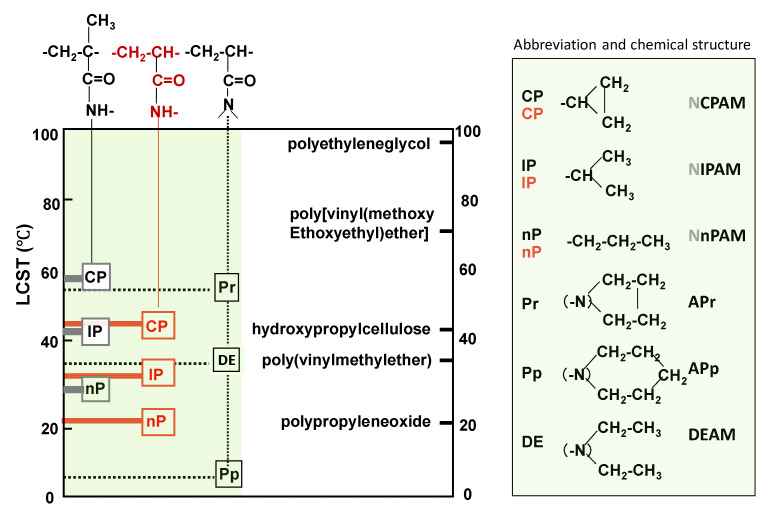
Volume phase transition temperaturs of acrylamide -derivative polymers and some other polymers.

Most of the data in Figure 5 were collected from the papers [43,44,45]. It shows VPTTs of a series of poly(acrylamide derivatives) and some other amphiphilic polymers.

First, the influence of three kinds of substituent propyl groups on the VPTT of the poly(*N*-substituted acrylamide)s is the focus. 

The order of VPTT_n-propyl_ < VPTT_isopropyl_ < VPTT _cyclopropyl_ would be explained by the order of the strength of the hydrophobic hydration of three polymers—in other words, the order of stability of water molecules nestling to polymer chains with each substituent. Normal propyl groups (n-propyl groups) in poly(*N*-n-propyl acrylamide) (PNnPAM) fluctuate relatively freely in their microenvironment and shake off more water molecules, nestling to polymer chains at lower temperature than other substituents (isopropyl and cyclo-propyl groups) systems, and as a result, the VPTT of PNnPAM becomes the lowest among the three. On the contrarily, compact cyclic propyl groups less destabilized water molecules nestling to polymer chains and allow them to keep the hydration state with polymer up to a higher temperature. Thus, poly(cyclo-propyl acrylamide) has the highest VPTT among the three.

Comparing VPTTs of poly(*N*-substituted acrylamides and methacrylamides), the former is lower than the latter. This suggests that the apparent hydrophilicity of polymers estimated from the chemical formula is not necessarily a suitable measure to decide VPTT. Instead of apparent hydrophilicity, the stiffness of a polymer main chain might be a more important VPTT-determining factor. The methyl group attached on the main chain increases the symmetry and stiffness of the main chain, decreases the fluctuation of the main chain, and keeps more water molecules nestling to polymer chains up to a higher temperature. Kokufuta et al. reported the endothermic enthalpy from the heating DSC curves increases in the order of poly(N-n-propylmethacrylamide) (PNnPMAM) > poly(N-n-propylacrylamide) (PNnPAM) ≈ poly(N-isopropylmethacrylamide) (PNIPMAM) > PNIPAM [45].

The VPTT of microgel can be shifted by changing the surrounding environment—for example, adding ingredients that compete with PNIPAM for hydration, such as acids, salts, hydrophilic compounds, and some biomolecules. Here, a coexisting system of typical hydrophilic polymers, polyvinylalcohol (PVA), and PNIPAM microgel is discussed. 

**Figure 6:**
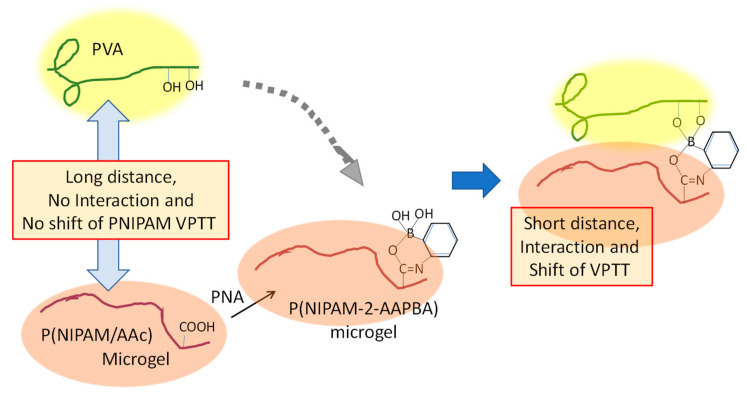
The forced pulling of PVA results in a decrease in VPTT f PNIPAM [46].

Contrary to expectations, the VPTT of PNIPAM microgel was little affected by the addition of PVA even if there was a high PVA concentration (the left of Figure 6). However, the VPTT of (PNIPAM core–P(NIPAM-co-2-acrylamido-phenylboronic acid (AAPBA))–shell) microgel, which was obtained by modifying a P(NIPAM–acrylic acid (AAc)) shell with 3-aminophenylboronic acid (APBA), was drastically reduced by the addition of a low concentration of PVA. It was proposed that the chemical adsorption of PVA chains onto the P(NIPAM-2-AAPBA) microgel shortened the distance between the PVA and PNIPAM chains and it enhanced the interaction between P(NIPAM-2-AAPBA) and PVA [46].

The next subject to be discussed is the sharpness of the VPT. The hydrodynamic diameter of PNIPAM microgel versus the temperature curve is expected to change discontinuously at the VPTT, but most often it does not. This fact may imply that transition is continuous. If so, the continuous behavior would be attributed to the possibility that the polymers exist at different states such as clustering, chain entanglement, different chain density at different positions from the core to the surface in the microgel, etc. [47]. 

To clarify this, Wu et al. carried out an interesting experiment. They prepared three PNIPAM materials—linear polymer, microgel, and bulk gel—and compared the temperature effect on volume phase transition among them [48]. The former two showed continuous transition. This was attributed to the inhomogeneous polymer chain length for the linear polymer and the inhomogeneous length sub-chains between the cross-linking points for the microgel. On the other hand, the discontinuous transition observed in the bulk gel was caused by the contribution of the bulk shear module and microscopic inhomogeneous shrinkage. 

The addition of a small amount (0.02% in monomer) of acrylic acid in the recipe of NIPAM precipitation polymerization was effective to realize a discontinuous volume transition of microgel. It was brought by loosening the polymer chain entanglement [49].

### 3.2. Size and Its Distribution of Microgels

From the viewpoint of the application of microgels, they are expected to be of suitable size and monodisperse as much as possible, except for rare cases in which a broad size distribution is preferable.

Microgels obtained by the general precipitation polymerization of N-substituted acrylamides have a diameter of 100 to 700 nm. If a smaller microgel is desirable, the addition of a small amount of surfactant gives good results.

Generally, products of precipitation polymerization are monodisperse in many cases, but the monodispersity of microgels formed in precipitation polymerization is not guaranteed. For example, the precipitation polymerization of vinyl chloride (VC) (monomer is used as the medium and as a non-solvent for poly-VC) results in the formation of distorted and uneven particles. This example teaches us the importance of moderate interaction among monomer–polymer mediums.

Monodisperse microgels are obtained when the following conditions are secured.

Particle formation should be ended in the early stage of polymerization, and after that, only existing particles should grow, and the existing particles should not suffer aggregation throughout the polymerization process. 

The above-mentioned conditions can be realized when the particles produced in the early stage of polymerization gain the stable state by mainly interparticle electrostatic repulsive force due to the charge generated from the initiator. Water molecules tightly bound to polymers may contribute to the further stabilization of particles. Since Pelton applied precipitation polymerization for PNIPAM microgel preparation, the details have become clearer about these points [1].

The size of microgels suitable for the application of interest must be selected considering the following factors. Smaller microgels have advantages including their quick response and dispersion stability. On the other hand, they have disadvantages in handling and recovering.

Varga et al. studied how to make the size of nanoparticles smaller [50]. The amount of emulsifier added and the temperature were shown to be important factors. 

On the other hand, some trials to enlarge microgels have been done by many researchers. The addition of salts in the microgel preparation stage gives a certain effect to get microgels over 1 micron, but it induces the random aggregation of microgels, resulting in broad size distribution [51]. 

Lyon et al. developed a novel method to prepare micron-sized multi-responsive microgels by temperature-programmed synthesis by precipitation polymerization with temperature change at a rate of 4.5 °C/min starting from 45 °C and finishing at 65 °C. This programmed procedure controlled the nucleation stage of polymerization and brought successful results in the formation of monodisperse larger microgels (e.g., microgels with 2.5 to 5 μm diameter) [52,53].

Ngai et al. sought to prepare a larger microgel by modifying the precipitation polymerization and obtained a monodispersed PNIPAM microgel of 4.7 μm diameter by combining semibatch and temperature-programmed surfactant-free precipitation polymerization [54,55]. Meanwhile, Suzuki’s group succeeded in forming microgels of 6.3 μm diameter by controlling the amount of cross-linker and its timing of charge [56].

These large microgels make it easy to observe their dynamic response to stimuli directly.

Differing from precipitation polymerization, membrane emulsification and microfluidic techniques were applied for the preparation of larger monodisperse microgels. Microfluidic devices allowed controlling microgels’ sizes as well as their internal structures [33].

NIPAM was polymerized and fabricated to microgel in dimethylsulfoxide (DMSO), which was was accomplished in a single-channel polydimethylsiloxane (PDMS) microfluidic device without surfactant [34]. The obtained PNIPAM microgels showed a sharp volume phase transition and high deswelling/swelling rate.

### 3.3. Cross-Linking Density and Its Distribution

The density and distribution of cross-links are the most important factors that determine the performance of microgels exhibiting volume phase transition. Generally, the density of cross-links is controlled by the amount of cross-linker such as methylene-bis-acrylamide (MBA). However, the precipitation polymerization of NIPAM with MBA is apt to form core–shell type microgels with a highly cross-linked core and slightly cross-linked shell. It is because highly polymerizable MBA localizes preferentially in the core and forms more cross-link structures there (Figure 1, top-left).

When the system contains an excess amount of MBA, the prepared microgels have a decanano scale non-thermoresponsive domain in the core due to too dense cross-links [57].

In order to prevent an uneven distribution of cross-linking points due to prior polymerization of the cross-linking agent, Richtering et al. attempted post-addition of the cross-linking agent and obtained a microgel having a uniform cross-linking density from the core to the surface [58].

If the polymerization system includes no cross-linker, PNIPAM particles prepared by the precipitation polymerization at temperatures above the LCST are expected to dissolve in the aqueous medium when the system is cooled down to room temperature (point A in Figure 3). However, it was found that the microgels obtained by cross-linker-free polymerization kept their figure even after one week in the aqueous medium at room temperature. This was attributed to cross-link structures existing unevenly in the surface layer of microgels. 

This unexpected result was attributed to the self-cross-linking of PNIPAM chains induced by initiator during the polymerization. Gao and Frisken analyzed the microgels that were prepared by cross-linker-free precipitation polymerization at 60–70 °C and confirmed that a certain amount of cross-links exist in the surface layer of microgels [59]. Cross-link formation without a cross-linker was attributed to chain transfer between the initiator molecule and polymer chain during microgel formation.

Richtering et al. argued that the cross-linked structure selectively generated on the microgel surface is due to hydrogen abstraction from the surface polymer by the initiator persulfate ion radicals. It is hard for the radicals to reach the core of the microgel, and consequently, the core has a lower polymer volume fraction than the surface in the swollen state [58]. Such microgels are prone to deformation, reflecting little cross-linking structure in the core. The easy deformation of less cross-linked PNIPAM microgel causes the fast spreading of microgel and lowers the surface tension [60].

Here, the works by Lyon’s group should be cited again. It was a trial to suppress the formation of undesirable cross-link formation in the surface zone of microgels. The polymerization at lower temperature by using UV irradiation and/or a redox initiator system was effective to achieve the purpose [52,53].

Precipitation polymerization using degradable cross-linkers is another way to prepare disposable microgel [61,62].

It would be worth mentioning that there is a large amount of inhomogeneity relating to uncontrolled radical cross-linking. The additional topological defects are dangling chain ends, cross-linker–cross-linker shortcuts, and chains forming loops, etc. [63,64].

### 3.4. Hybridization

Hybridization is a powerful means of adding functionality to thermosensitive microgels. The following are some examples of hybrid microgels in which inorganic and organic materials were incorporated simultaneously with or after the microgel preparation. 

The first example is a silica–microgel hybrid. Silica nanoparticles were decollated on a shell layer of P(NIPAM-co-dimethylaminoethyl methacrylate, methylchloride quaternized (DMC)) microgel. DMC rich in the shell of microgel promotes silica deposition [65]. The extent of silica deposition can be controlled with the reaction conditions. Sufficient deposition inhibited excess hydration swelling of PNIPAM.

Ballauff et al. prepared hairy PNIPAM particles and attached Ag nanoparticles near the root of hairs. The rate of catalytic reduction by the hybrid particle was investigated as a function of temperature [66].

ZnO particles were coated with PNIPAM hydrogel nanoparticles [67] in a three-step reaction. The first step was preparation of the PNIPAM hydrogel microgel (hydrodynamic diameter; 128 nm); the second was mixing PNIPAM hydrogel nanoparticles with ZnO (diameter: 160 nm), and the third involved coalescing the PNIPAM hydrogel and ZnO using electrostatic force. The final product, a ZnO core–PNIPAM shell, had a hydrodynamic diameter of 330–350 nm.

Inorganic/polymer nanocomposite SiO_2_–PNIPAM was synthesized through a one-pot approach in supercritical carbon dioxide (scCO_2_). All raw materials, NIPAM, vinyltriethoxysilane (VTEO), tetraethoxysilane (TEOS), initiator 2,2′-azobisisobutyronitrile (AIBN), cross-linker MBA, and hydrolysis agent acetic acid were introduced into one autoclave, and the parallel reactions of free radical polymerization and hydrolysis/condensation occurred simultaneously in the reaction mixture with scCO_2_ as a solvent [35]. The in vitro release simulation of the particles in situ impregnated with ibuprofen indicated that SiO_2_–PNIPAM composites, whose diameter was less than 100 nm, could improve the drug-releasing effect of the microgels as controlled drug delivery systems.

The next microgel has a sensing function. Phenylboronic acid has been used for glucose determination. A composite microgel with a function for glucose determination was developed [68]. The novel core–shell nanoparticles were synthesized by the self-assembly of a phenylboronic acid (PBA)-based block copolymer PNIPAM-block-poly(3-acryl-amidophenylboronic acid) (PNIPAM_136_-b-PAPBA_16_) and a fluorescent complex glucosamine-PNIPAM/Eu(III) (GA-PNIPAM)/Eu(III) based on the cross-linking between PBA- and GA-containing blocks. The nanoparticles can be tuned via thermo-induced collapse or glucose-induced swelling at appropriate pH and temperatures.

## 4. Physicochemical Characteristics of Thermosensitive Microgels

This section gives an overview of the physical properties that change discontinuously at the transition temperature. The transition temperature is the temperature at which the gel changes from the hydrated state to the dehydrated state. First, attention is paid to the change in surface tension. Next, the temperature dependence of electrophoretic mobility will be taken up.

### 4.1. Surface Activity and Adsorption of Microgels

Thermosensitive polymers are polymers whose hydrophilicity/hydrophobicity changes with temperature. Therefore, they exhibit temperature-dependent surface activity. Both the surface tension of PNIPAM aqueous solution and the interfacial tension of n-hexane/PNIPAM aqueous solution decreased with elevating temperature in the range from 16 to 31 °C. This phenomenon is explained by the increase in adsorption rate due to the dehydration and globulization of the polymer with increasing temperature. The interfacial tension of n-hexane/PNIPAM aqueous solution reaches the equilibrium value faster than the surface tension of PNIPAM aqueous solution [69].

Pelton et al. measured the dynamic surface tension of PNIPAM microgels with different MBA content at 25 °C and 40 °C [60]. The surface tension decreased due to the adsorption of the microgel on the surface. The lower the temperature and amount of the cross-linking agent, the faster the equilibrium value was reached.

The PNIPAM microgel’s interfacial properties in a dodecane/water interface was measured with a pendant drop tensiometer in the temperature range wider than Pelton’s—that is, from 20 to 45 °C. A V-shape of the surface tension versus temperature curve was obtained. The tension-decreasing part below VPTT was attributed to the dense adsorption of microgel layers and the increasing part above VPTT was attributed to adsorption of loosely packed PNIPAM microgels [70].

Ngai et al. also observed an anomalous interfacial tension minima of PNIPAM microgels around the VPTT of PNIPAM at the heptane–water interface. The authors considered that both dynamic and static parameters contributed to the observed interfacial tension minima. The PNIPAM microgel deformability dynamically dominated the microgel spreading at the heptane–water interface in the early states, while PNIPAM microgel packing and interactions dominated the final static equilibrium states [71].

A study of microgels’ behavior at interfaces is useful for designing Pickering emulsions. Ngai et al. examined the structure of micron-sized PNIPAM-based microgels at the decane–water interface and found that the microgels in alkaline condition are swollen and flattened anisotropically [72].

Two kinds of PNIPAM microgels having diameters of 250 and 760 nm were prepared, and the size dependence of the stability of Pickering emulsion was studied [73]. The bigger microgels induced an evolution from dispersed drops to strongly adhesive drops and flocculated emulsions. As the microgels size increases, their internal structure as well as the polymeric interfacial layer become more heterogeneous. The loss of a uniform dense layer favors bridging between neighboring drops, leading to flocculated and therefore less handleable emulsions.

**Figure 7:**
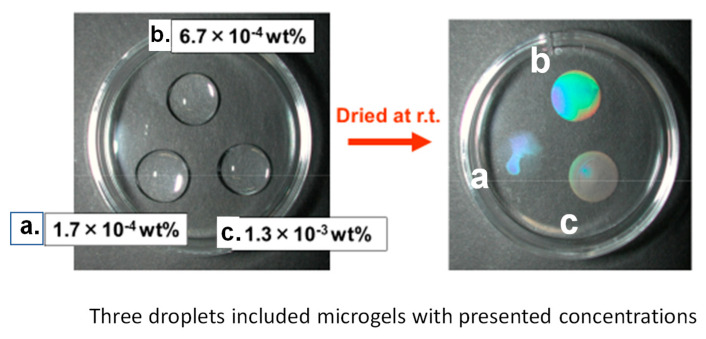
Structural colors observed in dried dispersions of PSt-core PNIPAM shell microspheres.

When a droplet of PNIPAM microgel dispersion with different concentrations (a: 1.7 × 10^−4^ wt %, b: 6.7 × 10^−4^ wt %, c: 1.3 × 10^−3^ wt %) was deposited on a substrate and dried up, the microgels remaining on the substrate formed an ordered array and emitted iridescent colors due to optical diffraction [74,75]. As presented in Figure 7, the trace made from a 6.7 × 10^−4^ wt % microgel dispersion presented a clear structural color in the whole area of the droplet trace. In contrast, the appearance of the trace that resulted from too concentrated dispersion was turbid, which suggested an overlapping of dried microgels in the trace, and that from sparse dispersion, a structural color appeared in a limited area.

Suzuki’s group elaborately investigated the course of drying of microgel dispersion droplets by using an optical microscope equipped with a charge-coupled device camera [76,77]. The transportation of the microgels to the air/water interface was due to the convection in the droplet. The adsorption of microgels to the air/water interface was strongly affected by the softness and surface activity of the microgels. In the course of drying, flattened soft microgels gathered at the air–water interface and formed a two-dimensionally ordered structure and emitted structural colors, which could be associated with interparticle distance [55,77]. 

Richtering et al. examined the structural ordering and phase behavior of charged PNIPAM microgels to confirm Gottwald’ theoretical prediction of a re-entrant–order–disorder transition (fluid–FCC–BCC–fluid). The phase behavior was observed as a function of concentration of loosely cross-linked ionic microgels [78]. 

The dispersion of PNIPAM microgels undergoes colloidal gelation, forming a three-dimensional sparse network-like structure in a hydrophobic and shrunken state above VPTT, despite their low particle volume. The effective surface charge density is expected to be a key factor governing the gelation and gel modulus [79].

### 4.2. Electrical Surface Phenomenon of Microgels

Cationic and anionic PNIPAM microgels were prepared by using cationic and anionic initiators. Their electrophoretic mobilities are almost zero below the LCST. It is because the microgels are highly swollen, and the ionic groups are buried in it. With increasing temperature above VPTT, the absolute value of electrophoretic mobility increased [80]. The microgel surface above VPTT had a clear shape, and the electrophoretic mobility was that of hard particles and pH-dependent. However, swollen microgel below VPTT had an unclear surface and gave pH-insensitive electrophoretic mobility [81].

DLVO (Derjaguin-Landau-Verwey-Overbeek) theory explains the stability of aqueous dispersions by quantitatively adding up electrostatic repulsion and the van der Waals attraction. Here, the electrostatic part of the DLVO interactions is discussed. Charged particles in an electrolyte solution possess electric potential, and when direct current is applied to the dispersion, the particles move at a rate (U) determined by the balance between the electric field strength (E) and viscous resistance (η) from the liquid. U/E is the so-called electrophoretic mobility (μ), which can be connected with the zeta potential (ζ) by Smoluchowski equation (Equation (1)). Here, ζ corresponds to the potential difference between particles’ slipping plane and a place far enough from the particle/water interface; therefore, it is a characteristic value to indicate the interparticle electrorepulsive force.

m = (ε_r_ ε_0_/η) ζ (1)

where ε_r_ and ε_0_ are dielectric constants of solution and in vacuum, respectively.

Ohshima pointed out that in the dispersion of soft-shell carrying particles, Equation (1) cannot describe their electrochemical properties [82,83]. This arises due to two reasons: (1) In the dispersion of core–shell particles, there is a layer of hydrated polyelectrolyte aqueous solution between the core polymer and electrolyte solution, and (2) differing from a rigid particle dispersion system, no slipping plane exists, and so, the zeta potential cannot be defined.

Ohshima carefully analyzed the result of electrophoretic mobility of core–shell particles and created a new formula, as shown in Equation (2), which can be applied for the dispersion of microgel diameter >> 1/κ (thickness of electric double layer). Equation (2) is characterized by two parameters, zeN (fixed charge density in polyelectrolyte) and 1/λ (softness parameter). The basic concept of Equation (2) is the existence of a polyelectrolyte zone which allows draining aqueous electrolyte solution in the soft particle dispersion, Equation (2).

(2) $$µ=\frac{\varepsilon_{r}\varepsilon_{0}}{\eta }\frac{\psi_{0}/K_{m}+\psi_{DON}/\lambda }{1/K_{m}+1/\lambda }+\frac{ZeN}{\eta \lambda^{2}}$$

where *Ψ*_0_ and *Ψ*_DON_ are the surface potential and Donnan potential, respectively, *κ_m_* is the Debye–Huckel parameter for the polyelectrolyte layer, *λ* equals (*γ*/*η*)^1/2^, where *γ* is a frictional coefficient, *η* is viscosity, *λ* characterizes the degree of friction exerted on the liquid flow in the polyelectrolyte layer, and 1/*λ* corresponds to the softness of the polyelectrolyte layer. Substituting 1/*λ* = 0 into Equation (2) yields Equation (1); that is, it means that the rigid particle’s surface directly faces the aqueous solution of electrolyte.

**Figure 8:**
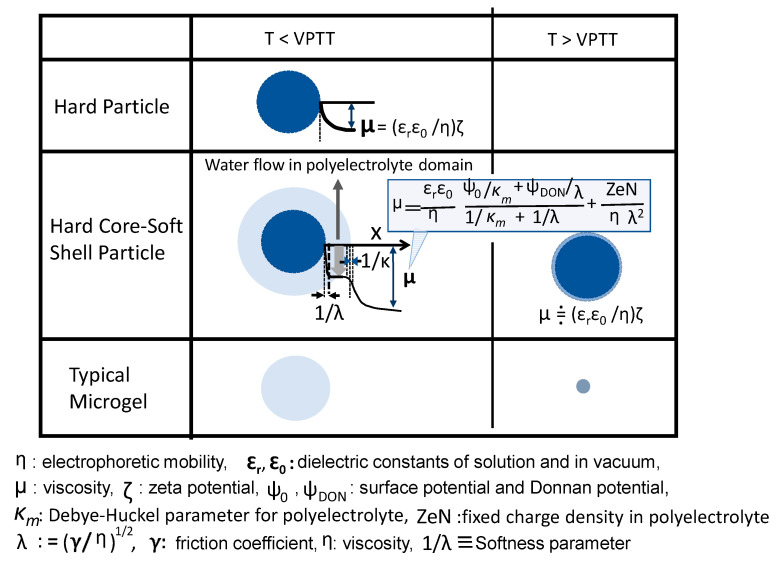
Colloid and surface chemistry of microparticles (Equation (2)).

Figure 8 shows three kinds of microparticles with their surface electric property below and above the VPTT. The electrochemical phenomenon of the point symmetry microgels having unclear surfaces is difficult to analyze, and there are only a few reports about them.

Oshima et al. applied this theory to the data of electrophoretic mobility of two kinds of PSt core–PNIPAM shell particles, one was negatively charged core–negatively charged shell particles, and the other was negatively charged core–nonionic shell particles [84]. The zN values of two particles and the 1/*λ* of the latter decreased with temperature, but the 1/*λ* of the former was independent of temperature.

Sounders group prepared non-charged PNIPAM (core)–charged PNIPAM (shell) microgels and analyzed their temperature-dependent hydrodynamic radius and electrophoretic mobility referring to Equation (2) [85]. The electrophoretic mobility versus temperature curve had an inflection point at a higher temperature than that in the hydrodynamic radius versus temperature. The results suggested that the core and shell responded in sequence as follows: (1) collapse of nonionic core, around 25 °C, (2) collapse of ionic shell, and (3) final collapse of core around 50 °C.

Pichot et al. applied Equation (2) to their PSt-core/PNIPAM-shell particles and recognized that Equation (2) worked at high electrolyte concentration but did not always hold at low electrolyte concentration [86]. Other researchers also confirmed the validity of Equation (2), but some questions were raised regarding draining in the polyelectrolyte layer [87,88].

Varga et al. investigated the draining behavior of the inner part of microgels and insisted that the contribution of the inner part to electrophoretic mobility in low ionic strength was little [89].

Ngai et al. studied the dependence of electrophoretic mobility and particle size of PNIPAM microgels on temperature, referring to the spherical electrolyte model. Results were consistent with what the theory suggested [90].

### 4.3. Dispersion and Aggregation of Microgels

Microgels are used as environmentally responsive emulsifiers. The following section compares the suitability of amphiphilic polymer and microgels as emulsifiers. The materials used were a poly(NIPAM-co-MA) microgel and low molecular weight PNIPAM, and the emulsions tested were the octanol/water system. Their ability was compared at different pHs and temperatures. The charged microgel demonstrated usefulness as a stimuli responsive stabilizer for emulsions [91]. Emulsification with high shear rates allowed the preparation of both w/o and o/w emulsions, but with low shear rates, o/w emulsion was preferably obtained. The stability of emulsion prepared with poly(NIPAM-co-MA) increased with increasing pH and lowering temperature [92].

The following papers discuss the aggregates or clusters of microgels, in which microgels were used as building blocks. Such kinds of materials hold great promise as tools that can quickly treat a large amount of reactants. 

The gelation of PNIPAM microgel dispersions is not brought by temperature change if no salt is added, even at temperatures well above the VPTT. However, at high ionic strength, microgels aggregated to form microgel assembly upon heating. The effect of NaCl concentration and temperature were dominating factors for gelation. The gelation temperature was slightly higher than the corresponding VPTT [93].

A new type of microgel cluster was proposed. It was prepared by clustering the PNIPAM satellite microgels (B) around the oppositely charged core particles (A). The resulting clusters, AB_4_, showed different dispersion behavior above and below the VPTT of B [94]. 

A novel type of microgel-cross-linked hydrogels (MCG), which are constructed with poly(NIPAM-co-acrylic acid) (P(NIPAM-co-AAc)) microgels as building blocks, are featured with both a fast response rate and large volume change ratio to environmental temperature stimuli. The proposed MCG hydrogels are attractive for developing efficient stimuli-responsive smart sensors, actuators, bio-separation absorbents, and so on [95].

The Weitz group developed quickly responsive hydrogel scaffolds that consist of a 3-dimensional colloidal network structure. In this proposal, poly(NIPAM-co-AAc) microgels and copolymers dissolved in microgel dispersion were used as a building block and a binder, respectively. The volume change of the scaffolds in response to temperature change was tuned by the cross-link density of the microgel. The release profile of a model drug was also regulated successfully by cross-link density [96].

Now, the stability of the microgel dispersion is discussed using the PSt core-poly(App + APr) shell particles as an example [38]. 

The stability of the dispersion is controlled with competing two forces—that is, electric repulsive force and hydrophobic attractive force. The former is controlled by the effective charge of microgels and the latter is controlled by interparticle cohesive force. In other words, the stability of microgels having VPT is affected by salt concentration and temperature.

**Figure 9:**
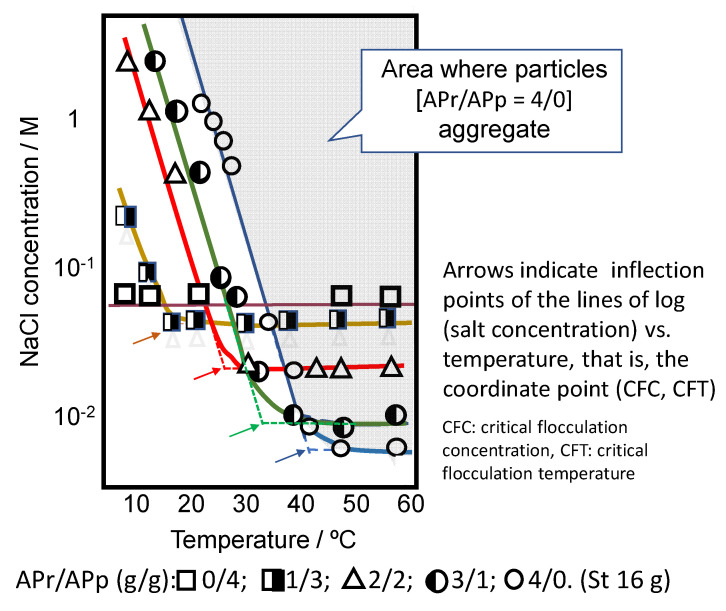
Dependence of stability of PSt-core–APr/APp-shell particles on temperature and NaCl concentration.

Temperature controls the extent of the hydrogen bond of polymers in microgel and salt concentration controls the electrorepulsive force among microgels. Figure 9 shows the boundary of stable and unstable dispersion of PSt-core–APr/APp copolymer-shell particles as a function of salt concentration and temperature, which was obtained by the observation of the appearance of core–shell particles dispersion at different salt concentrations and different temperatures. A peculiar inflection point is recognized in each system in the figure. We defined the coordinates of the inflection point as (CFC (critical flocculation concentration), CFT (critical flocculation temperature)) for each system and replotted the data on the figure of log (salt concentration–CFC) versus (temperature–CFT). The curves for 4 systems of APr/App = 1/3 to 4/0 were almost collected into one master curve.

## 5. Remaining Problems on Functional Microgels and Strategies for Solving Them

**Figure 10:**
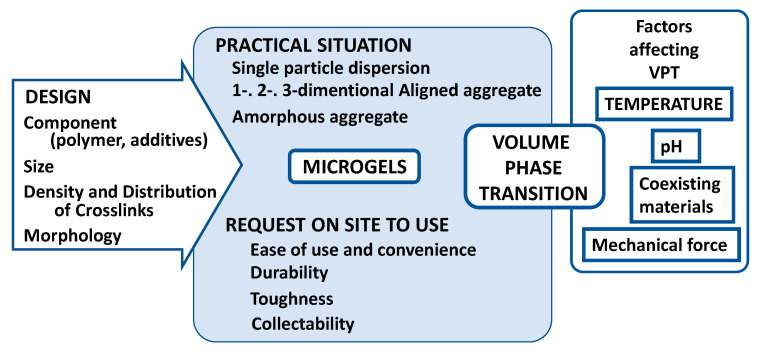
Items to be considered from synthesis to application of practical microgels.

The final section of the review may address the fantastic applications of microgels exhibiting volume phase transition. However, outlining all the papers featuring the application of microgels would be rambling. Therefore, in this section, we will summarize the points to be noted when considering various applications of microgels. Figure 10 might be useful for consideration.

Up to now, we have almost acquired the technique of producing particles with the desired size. On the other hand, little technology has been established to eliminate heterogeneity in the internal structure, such as the bias of cross-linked structure, distribution of inter-cross-link chain length, uncontrollable dangling chain ends, etc. Radical polymerization is used for the preparation of microgel so far. However, polymer synthesis that does not rely on free radical polymerization, such as living polymerization, monomer order-regulated polymerization, etc. may give an answer to this problem. The design and use of cross-linking agents might be the key to the future of functional microgels. In the research of bulk gels, new technologies such as interpenetrating polymer network [97,98], nanocomposite gel [99], tetra-gel [100], and slide ring gel [101] have been applied to get the gels with a characteristic nanostructure.

As mentioned in Section 3, the smaller the particle, the faster the reaction. However, the smaller the particle, the more troublesome the treatment is another truth. The assembling of microgels to a three-dimensional structure has been tried as a method to overcome this trade-off [94,95,96]. Rosary or necklace-shaped microgel aggregates are also candidates.

The development of instrumental analysis for evaluating the performance of microgels is remarkable, and the progress of technology to make full use of it is also highly evaluated.

For example, a high-speed atomic force microscope can set up an environment where real-time phenomena can be observed [57]. 

The autocorrelation function required for the DLS of microgel suspensions with different concentrations is specific to each concentration region. The data from the dispersion of low concentration provide information on the colloidal character—that is, the hydrodynamic size of the microgels. Furthermore, the data from higher concentration dispersion provides the information on gel network structure [102]. A worldwide collaboration of researchers sharing high-performance equipment would encourage the construction of a new era of microgels.

## 6. Afterword

It has been 100 years since macromolecules were recognized by academic societies. In less than 40 years, thanks to the efforts of researchers in polymer science, colloid science, surface chemistry, and biophysics, microgels have become a major research theme.

Among them, microgels that exhibit a volume phase transition have attracted many researchers and the research have given us rich fruits, a part of which was presented in this review. However, some say that it still contributes still too little to society despite the depth of research. From now on, microgel researchers will enter a new era of gel science and technology, seeking a greater relationship with society.
